# Supplementary figures and images for: Angiopoietin-2 and Angiopoietin-like Proteins with a Prospective Role in Predicting Diabetic Nephropathy
Source: Biomedicines. 2024 Apr 24;12(5):949. doi: 10.3390/biomedicines12050949 (PMC11118931; doi:10.3390/biomedicines12050949)

Figure S1

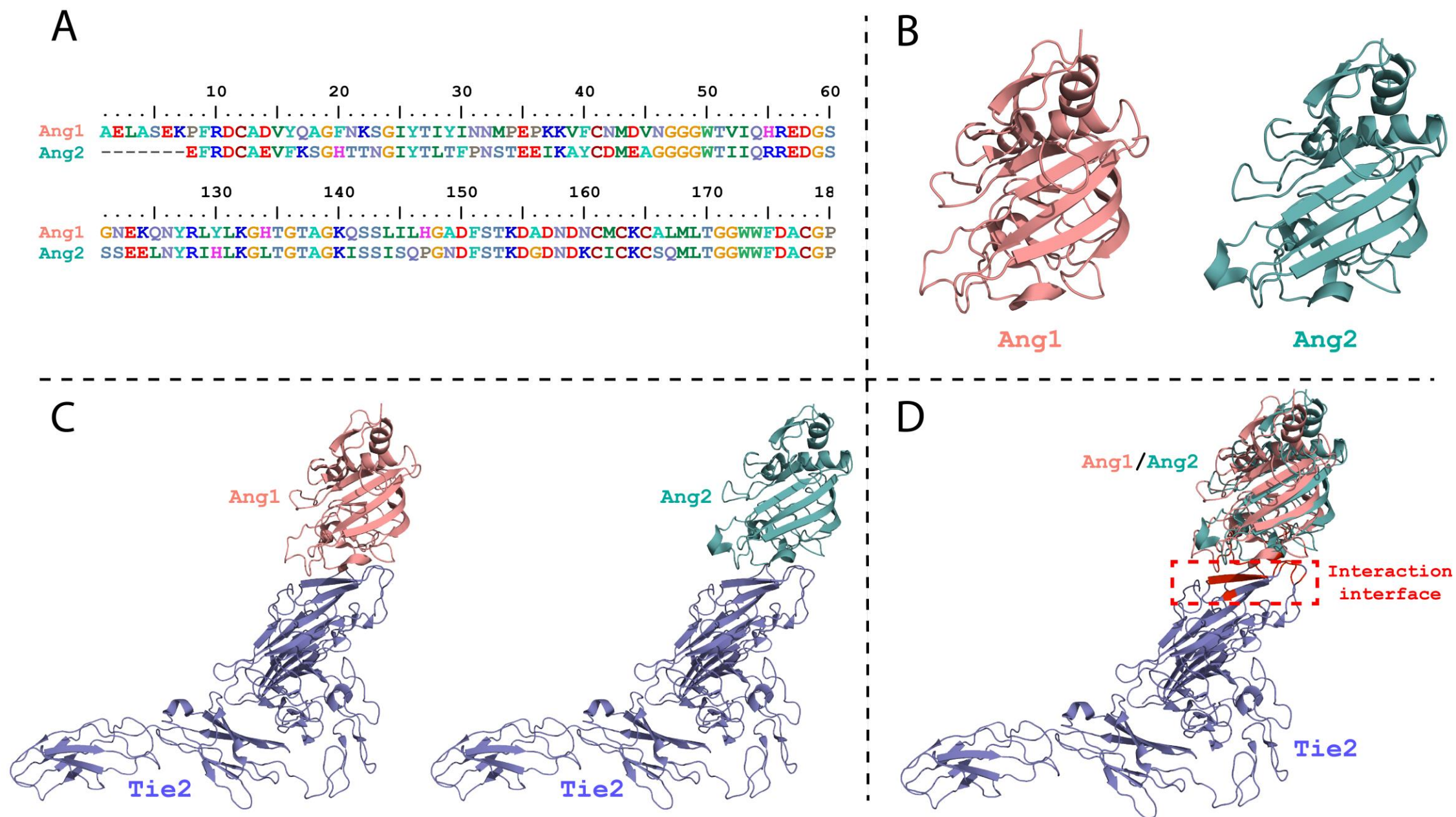

**Figure S.2**

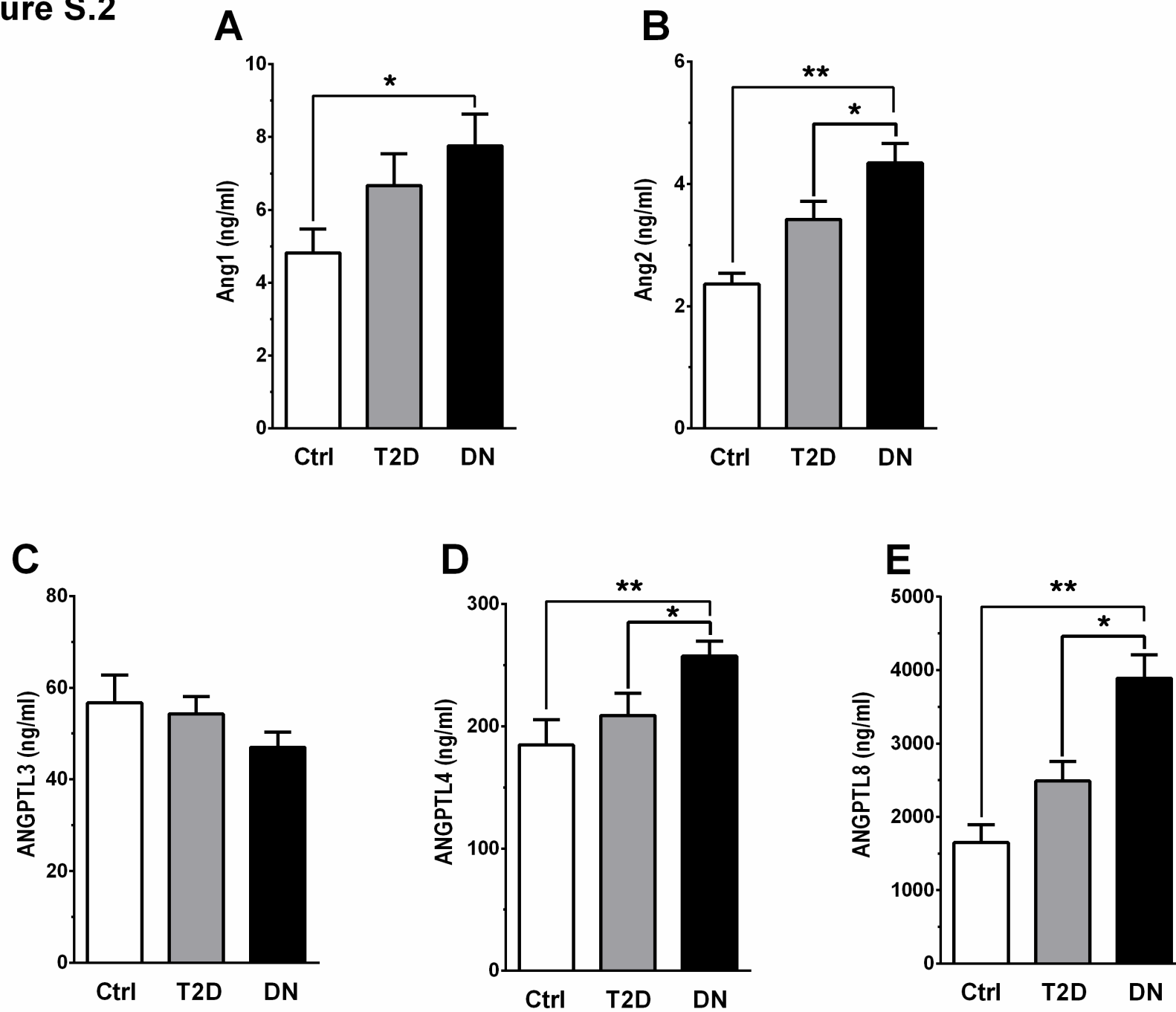

Supplement: Supplementary file 1 [file biomedicines-12-00949-s001.zip › biomedicines-2957839-supplementary.pdf]
